# Exposure Characterization of Haloacetic Acids in Humans for Exposure and Risk Assessment Applications: An Exploratory Study

**DOI:** 10.3390/ijerph16030471

**Published:** 2019-02-06

**Authors:** Shahid Parvez, Jeffrey L. Ashby, Susana Y. Kimura, Susan D. Richardson

**Affiliations:** 1 Department of Environmental Health Science, Indiana University Fairbanks School of Public Health, 1050 Wishard Boulevard, Indianapolis, IN 46202, USA; jlashby@iu.edu; 2 Department of Chemistry and Biochemistry, University of South Carolina, 631 Sumter St., Columbia, SC 29208, USA; s.kimurahara@ucalgary.ca (S.Y.K.); richardson.susan@sc.edu (S.D.R.); 3 Currently at the Department of Chemistry, University of Calgary, Calgary, AB T2N 1N4, Canada

**Keywords:** disinfectant byproducts, haloacetic acids, monochloroacetic acid, trichloroacetic acid, exposure assessment, risk assessment of haloacetic acids, pregnancy outcomes

## Abstract

Disinfected water is the major source of haloacetic acids (HAAs) in humans, but their inter- and intra-individual variability for exposure and risk assessment applications is under-researched. Thus, we measured HAAs in cross-sectional and longitudinal urine and water specimens from 17 individuals. Five regulated HAAs—mono-, di-, and trichloroacetic acid (MCAA, DCAA, and TCAA) and mono- and dibromoacetic acid (MBAA and DBAA)—and one unregulated HAA—bromochloroacetic acid (BCAA)—were measured. Urinary DCAA, MBAA, DBAA, and BCAA levels were always below the limits of detection (LOD). Measured levels and interindividual variability of urinary MCAA were higher than urinary TCAA. Longitudinal urinary specimens showed MCAA levels peaked in after-shower specimens, while TCAA levels remain unchanged. Correlation between urinary MCAA and TCAA was moderate but statistically significant. The prevalence of MCAA and TCAA in urine suggest they can be considered as biomarkers of HAA. Peak urinary MCAA in post-shower specimens suggest MCAA captures short-term exposure via dermal and/or inhalation, while urinary TCAA captures long-term exposure via ingestion. However, further research is warranted in a large pool of participants to test the reliability of MCAA as exposure biomarker.

## 1. Introduction

Haloacetic acids (HAAs) are the second most prevalent group of disinfection byproducts (DBPs) in disinfected water [1,2,3]. They are formed by the reaction of disinfectants (chlorine, chloramine, chlorine dioxide, and ozone) with natural organic matter and bromide [4]. They account for more than 25% of the total halogenated DBPs formed in chlorinated drinking water [5,6]. The U.S. Environmental Protection Agency (EPA) regulates five HAAs (monochloroacetic acid, MCAA; dichloroacetic acid, DCAA; trichloroacetic acid, TCAA; monobromoacetic acid, MBAA; and dibromoacetic acid, DBAA). Although, their combined maximum contaminant level permissible in U.S. drinking water is 60 µg/L, their levels vary substantially in drinking water, due to differences in source water characteristics (dissolved organic carbon concentrations and composition of natural organic matter-NOM), seasons (summer vs. winter), disinfectant type (e.g., chlorine, chloramine, chlorine dioxide, and ozone), and disinfectant doses [3,6,7,8,9].

HAAs are mutagenic, cytotoxic, genotoxic, teratogenic, and carcinogenic [10,11,12,13,14,15]. Recent research has shown that HAAs can affect pyruvate dehydrogenase activity and disrupt cellular metabolism [16]. Chlorinated HAAs (e.g., DCAA and TCAA acid) induce oxidative stress in rats by reducing glutathione (GSH) synthesis [17]. Monohaloacetic acids (including chloro-, bromo-, and iodoacetic acid) can cause genomic DNA damage in Chinese hamster ovary cells, and inhibit follicle growth and steroidogenesis in mice ovary [13,18,19,20].

Millions of people worldwide, including sensitive subpopulations (e.g., pregnant women and children) with potential increased risk of experiencing reproductive and developmental health effects, consume disinfected water [21,22,23,24,25,26,27]. Most population-based epidemiologic studies use aggregated compliance data on five-HAAs for assessing their association with adverse reproductive outcomes. However, only a few studies reported the use of individual HAAs, especially urinary measures and reproductive health risk, which may provide more conclusive evidence of health risk [28]. For example, a retrospective cohort study from Central Arizona reported that the water concentrations of DCAA > 18 µg/L and TCAA > 17.8 µg/L increase the risk of small for gestational age (SGA), and the consumption of DBAA (>5 µg/L) and DCAA (>8 µg/L) later in pregnancy (weeks 33–40) elevate the risk of low birthweight and intrauterine growth restriction [29]. Similarly, a study from Maryland county (1998–2002) estimated the exposure of five-HAAs using drinking water proxy, and reported an increased risk of intrauterine growth retardation. There seemed to be an elevated risk in third-trimester with water BCAA > 0.7 µg/L, DBAA > 0.8 µg/L, and DCAA > 14–15 µg/L [30]. Epidemiologic evidence with urinary HAAs is limited, as only two reproductive epidemiologic studies have evaluated them as exposure biomarkers. These studies reported the associations of urinary TCAA (>10 µg/L) with mean birthweight reduction [31], and SGA [32], but corresponding drinking water measurements were absent, preventing a comprehensive assessment of the relationship between drinking water and urinary HAAs. Both studies used the information on the water use activities (e.g., daily water intake, bottled water use, shower and bath habits, and time and duration of swimming in pools) from participants to develop exposure estimates from different routes.

HAA exposure via measurements in urine and residential drinking water that include determinations of their exposure variability will help to identify the reliable biomarkers of exposure for reproductive epidemiologic studies. In the absence of biomarker data, exposure scientists, and risk assessors rely on regulatory compliance data in drinking water as a surrogate measure of HAA exposure in humans, assuming that individual HAAs continue to behave in the same way once consumed. However, the composition of individual HAAs changes with water distribution systems, disinfectant type, and nature of NOM, and the sum total of the five regulated HAAs for compliance use does not account for differences in individual HAAs. It also does not account for toxicokinetic mechanisms or intra- and interindividual variability due to behavior differences in water use activities and water source characteristics, which could potentially change HAA exposure [1,6,28,33,34].

Parvez et al.’s (2017) study on short-term (weekly and biweekly) exposure assessments of regulated HAAs in drinking water showed high exposure variability. Thirty-five per cent of water specimens had HAA levels above the regulatory limit, demonstrating that compliance data does not capture short-term exposure variability, which is essential to determine their correlation with reproductive health risks [28]. Moreover, the compliance-based drinking water data do not account for differences in exposure routes, multiple water sources (e.g., bottle water, office, home, and gym), or variability in HAA composition between urinary and drinking water metrics. In spite of these challenges, health scientists still use compliance data on HAAs as an exposure proxy for predicting the reproductive health risk. For this reason, several epidemiologic studies were susceptible to exposure misclassification and remain inconclusive about the exposure risk of HAAs on reproductive health [22,24,25,26,27,35,36,37,38]. Biomonitoring may offer more reliable exposure measures of HAAs and minimize exposure misclassification for more conclusive epidemiologic investigations and risk assessment. However, the absence of well-characterized exposure data on urinary biomarkers of HAAs poses a challenge to design more reliable reproductive epidemiologic investigations.

Only a few studies have measured urinary excretion of HAAs [39,40,41,42,43]. Moreover, studies on inter- and intra-individual exposure variability and sensitivity to exposure routes are limited. TCAA is frequently detected in urine because of its long half-life (1–6 days) [42,43], and reported as a biomarker of HAAs, but its interindividual and intra-individual variability is not fully known in humans. A few studies measured urinary HAAs in swimmers and pool workers, as well as in people after domestic activities (e.g., showering, bathing, and washing dishes and clothes), which essentially captured high exposure events, but did not capture baseline exposure levels [40,41]. Also, relying on TCAA as the only HAA biomarker may not represent cumulative exposure of all individual HAAs. Currently, no study has evaluated whether urinary MCAA can be used as a reliable exposure biomarker. This is important because MCAA is more cytotoxic and genotoxic than DCAA and TCAA [19,20,44]. Therefore, an exploratory human biomonitoring study was designed, and participants were recruited from two water systems to determine HAA composition in urine and the corresponding drinking water, compare inter- and intra-individual exposure variability, and investigate potential correlations across urinary biomarkers and between urinary and drinking measures. 

## 2. Materials and Methods 

### 2.1. Participant Enrollment and Water Supplies

The Indiana University Institutional Review Board (#1412990740) approved the study. The participants (age ≥ 18 years) were recruited and consented from two sites that are located in the midwestern United States (Site A) and southeast United States (Site B). The participants were contacted via email blast and local announcement. The participation in the study was completely voluntary and participants did not receive payment for their participation in this study. The raw water source at Site B has relatively high levels of bromide compared to Site A. High bromide levels are typically found in source waters near the coast, where >50% of the U.S. population lives. Bromide reacts with NOM and disinfectants to form brominated disinfection byproducts (DBPs) [3,5,45,46], which are much more cytotoxic, genotoxic, mutagenic, and carcinogenic than chlorinated DBPs [10]. While both water systems use chlorine or chlorine dioxide as the primary disinfectant followed by chloramine for secondary disinfection, two sites had reportedly no difference in temperature and rainfall. These variables influence disinfectant byproduct formation. High temperature (e.g., during summer) and high rainfall influence HAA formation [6]. Both sites reported moderately low temperature (Site A 26 F and Site B 44 F) and rainfall (Site A 0.2 cm and Site B 0.38 cm rain) during the sampling period, thus we expect no changes in HAA formation during sampling duration. Information on doses of disinfectants, residual chlorine in the distribution systems, and pH of treated source water were not available.

### 2.2. Urine and Drinking Water Sampling

After the initial recruitment and consent process, each participant was provided training on the urine and water collection procedures and recording of three water-linked exposure activities, e.g., showering, bathing, and dish washing. The reporting of information on frequency and amount of water intake, sources of water, consumption of beverage, and physical activities (e.g., exercise) was voluntary. However, participants were advised to drink unfiltered tap water and avoid alcoholic beverages 24-h prior and during the sampling period. Each specimen was labeled with participant and specimen identification numbers and time of sampling. In case of longitudinal specimens, participants were additionally instructed to label specimen collected after shower because of differences in shower time across participants. Cross-sectional urine (single specimen per participant) specimens were collected from 17 participants residing at Sites A and B, while 24-h longitudinal urine (three to four specimens per participant) specimens were collected from three participants residing at Site A (Table 1). 

Twenty-four-hour urine specimens were collected at different time intervals: first morning void (MV), post-shower (PS), and evening or before going to sleep in the evening (NT). Same-day unfiltered tap water specimens were also collected from each individual’s home. Participants were provided a sampling kit and a cooler with dry ice for temporary storage. The time of specimen collection and exposure-linked activities were recorded. The specimens were processed per the laboratory protocol and shipped to a state-approved analytical laboratory for analysis of HAAs (Brookside Laboratory, New Bremen, OH, USA). It is important to note that same-day urine and drinking water specimens were collected from each individual, but the day and time of specimen collection varied across participants. All the specimens were temporarily stored at a central facility at Site A and Site B before they were shipped to the analytical laboratory. All the specimens were shipped to the analytical laboratory within 24 h and analyzed within one week of collection per laboratory protocol.

### 2.3. Laboratory Measurements 

The water specimens were measured for total organochlorine (TOCl), total organic bromine (TOBr), and total organic iodine (TOI), using a total organic halogen (TOX) analyzer (Mitsubishi Chemical Analytech, Chigasaki, Japan; Cosa Xentaur, Yaphank, USA) coupled with ion chromatography using a Dionex 1600 ion chromatograph (Dionex, Sunnyvale, CA) or an inductively coupled plasma-mass spectrometer (Finnigan ELEMENT XR, Thermo Electron Corporation) [4]. Urine and drinking water specimens were analyzed for HAAs using EPA Method 552.3, which uses a gas chromatography-electron capture detection [47]. This method for TCAA was more sensitive (limit of detection, LOD, 1µg/L) than the gas chromatography coupled mass spectrophotometry method reported in the French study with LOD = 10 µg/L [32,48]. All urine measurements were adjusted for creatinine in order to address variability in the dilution of urine [49].

### 2.4. Data Analyses

The data analysis was performed to determine interindividual and intra-individual (24-h) variability and correlation analysis between urine biomarkers to evaluate the feasibility of using them as reliable exposure biomarkers of HAAs. Numeric variables were summarized by mean (standard deviation), and median (interquartile range). HAA levels were compared using the nonparametric Mann–Whitney test. Due to small number of specimens and non-normal nature of the generated data, Mann–Whitney test is preferred over Student’s *t*-test [50,51]. Levels of individual HAAs below LOD were imputed with LOD/2 as conventional practice for handling small dataset with non-normal distribution [52]. Although, other advanced statistical techniques such as Cohen’s maximum likelihood estimation or Bootstrap are preferred over standard replacement techniques, these techniques require the data to be normally distributed without the nondetects. The reported LODs for DCAA, TCAA, DBAA, and BCAA were 1 µg/L, and MCAA was 2 µg/L. Two-tailed *p*-values <0.05 was considered significant. Spearman’s correlation coefficients were determined. All data analyses were performed using R (v 3.1.2) (R Foundation for Statistical Computing, Vienna, Austria) and Microsoft Excel® 2013 (Microsoft, Sacramento, CA, USA).

## 3. Results

### 3.1. Comparison of Two Drinking Water Systems Based on Total Organic Halogen

**Figure 1:**
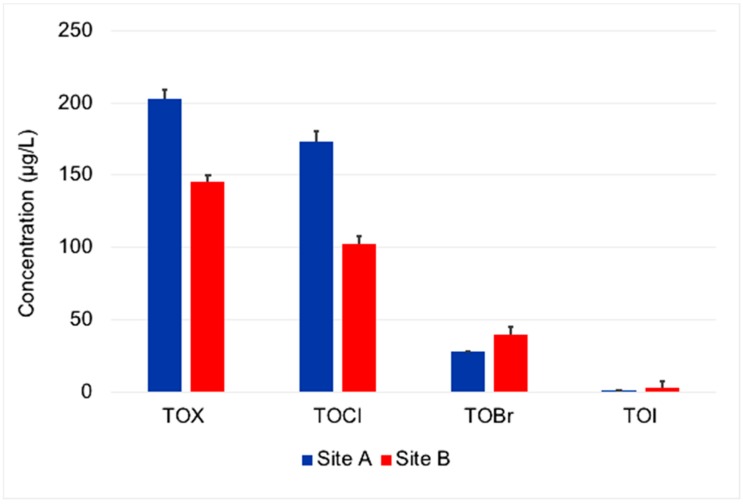
Comparison of total organic halogen in drinking water systems located at Site A and Site B. Legend: Three specimens were collected from each site. The error bars indicate standard deviation in the measured levels. TOX and TOCl levels were statistically different between the two sites (TOX *p* = 0.01; TOCl *p* = 0.0006).

The analysis of drinking water specimens from the two water systems showed significant differences in TOCl and TOX (Figure 1). TOX (Site A: 202 µg/L vs. Site B: 145 µg/L; *p* = 0.01) and TOCl (Site A: 173 µg/L vs. Site B: 103 µg/L; *p* = 0.0006) levels were substantially higher at Site A when compared to Site B. TOBr levels at Site B were higher than at Site A but the difference was statistically insignificant (Site A: 28 µg/L vs. Site B: 40 µg/L, *p* = 0.13). Similarly, TOI levels in the two water systems were negligible and were not statistically different (Site A: 1.44 µg/L vs. Site B: 2.52 µg/L, *p* = 0.22).

### 3.2. HAA Concentrations in Urine and Drinking Water

The raw data on all individual HAAs measured in urine and drinking water specimens are provided in the supporting data section (Table S1). Participants from the two water systems had comparable levels of urinary HAAs (Table 2). TCAA was the most prevalent HAA (89% detection rate), followed by MCAA (44% detection rate). Other individual urinary HAAs (i.e., DCAA, MBAA, DBAA, and BCAA) were always below the LOD. The concentrations of MCAA ranged from 1.0 to 12.6 µg/L, and TCAA ranged from 0.5 to 7.6 µg/L. The analysis of interquartile range (IQR) showed significantly higher levels and exposure variability in urinary MCAA than urinary TCAA (*p* = 0.04). However, the comparison of urinary MCAA and TCAA from two sites showed no significant differences (MCAA: *p* = 0.71; and TCAA: *p* = 0.47). 

The composition of individual HAAs in drinking water was very different from that of the urinary HAAs. MCAA was never detected in the drinking water specimens, yet it was often observed in the participants’ urine. While DCAA, TCAA, and unregulated BCAA were detected in all water specimens (Table 3), of these, only TCAA was found in the corresponding urine specimens. Mean levels of DCAA (15.9 ± 4.9 µg/L) were higher than TCAA (6.6 ± 3.4 µg/L) and BCAA (4.2 ± 0.7 µg/L). DCAA and TCAA showed site-specific differences in their levels (DCAA *p* = 0.01), while TCAA and brominated HAAs e.g., DBAA and BCAA showed insignificant site-specific differences in their levels (TCAA *p* = 0.66, DBAA *p* = 0.10, and BCAA *p* = 0.13).

### 3.3. Correlation of Urinary and Drinking Water TCAA and MCAA

The analysis of scattered plots showed monotonic (nonparametric) relationships between urinary MCAA and TCAA; hence the Spearman’s rank correlation coefficient (r) was used [53]. It shows a moderate but statistically significant correlation (r = 0.45; *p* = 0.04). The Spearman’s rank correlation between urinary and drinking water TCAA was moderate but statistically insignificant r = 0.52; *p* = 0.07). The correlation between urinary and drinking water MCAA could not be established, as none of the drinking water specimens had detectable levels of MCAA. Similarly, the correlations of other individual HAAs could not be established in absence of detectable levels in one or another matrix.

### 3.4. Longitudinal (Intra-Individual) Exposure Variability of Urinary MCAA and TCAA

**Figure 2:**
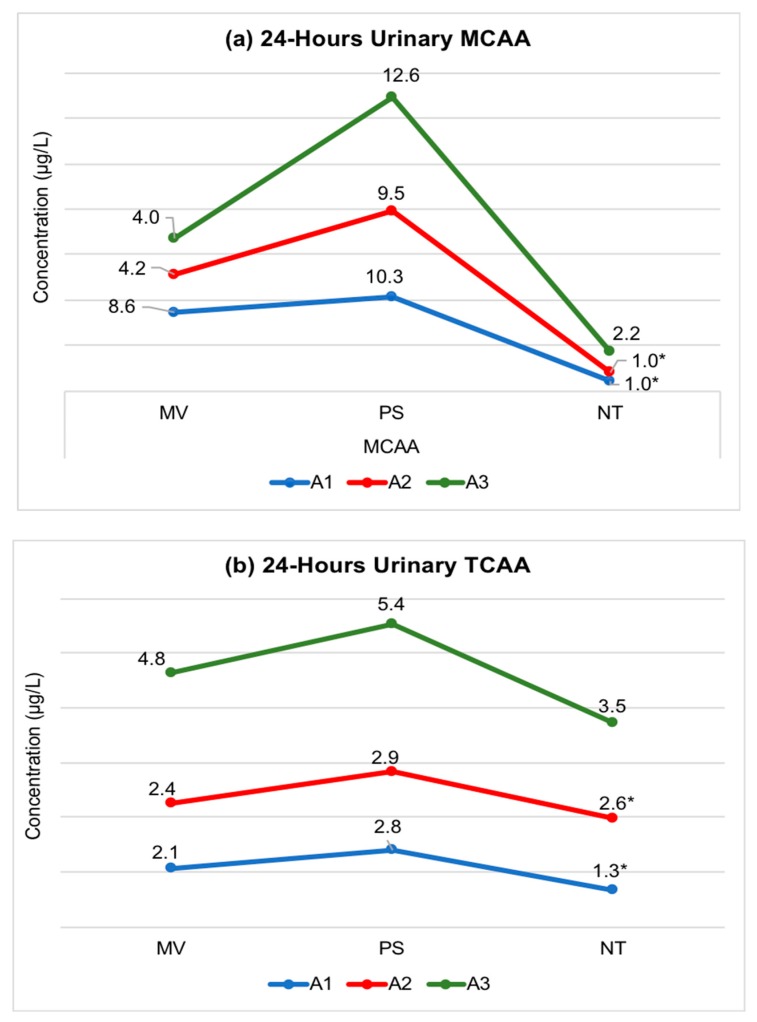
Intra-individual (24-h longitudinal) exposure trend of urinary MCAA and TCAA levels. Legend: Graphs show MCAA (Figure 2a) and TCAA (Figure 2b) concentration change in urinary specimens collected from three participants A1, A2, and A3. Specimens were collected at morning void (MV), post-shower (PS), and nighttime (NT). * represent mean values derived from participant A1 and A2 who provided duplicate NT specimens, while other values are derived based on single specimen analysis. All individual level data are provided in the supporting material Table S3.

Trend analysis of intraday variability of urinary MCAA and TCAA is important for finding the baseline and peak exposure levels. Both MCAA (ranged from 1–12.6 µg/L) and TCAA (ranged from 1.3–5.4 µg/L) were detected in the urine specimens and showed high exposure variability (Figure 2). MCAA levels peaked in PS specimens, while MV and NT specimens tended to have low levels. Most urine specimens collected within three hours of shower consistently showed high levels of MCAA. Trend analysis of TCAA levels show moderate increase in PS levels but the degree of increase was not as prominent as PS levels of MCAA. Due to three specimens per participant (i.e., each at MV, PS, and NT), and skewed dataset, no statistical test was performed to determine whether MCAA and TCAA levels were significantly different for participants across different time points.

## 4. Discussion

The use of two different sites in this study allowed the comparison of site-specific differences in composition and exposure variability of individual HAAs. Sites A and B both use chlorine or chlorine dioxide disinfectant followed by chloramine for disinfection but show significant site-specific differences in TOX and TOCl levels. However, no significant site-specific differences were observed for TOBr and TOI. Site A contained higher amounts of chlorinated HAAs than Site B, which contained slightly higher levels of TOBr and brominated HAA species (e.g., BCAA). The difference in TOX and TOCl levels at the two sites is likely due to differences in amount of natural organic matter and water treatment conditions at the two sites, such as chlorine dose, reaction time, and residual time [6,28,34,54]. Contrary to TOX and TOCl, the measured levels of individual HAAs (except DCAA) in drinking water showed no significant site-specific differences. This suggests that significant differences in TOX, TOCl, and TOBr levels at the two sites are mostly due to other halogenated DBPs, possibly including unmeasured HAAs. These halogenated DBPs include trihalomethanes, haloacetaldehydes, haloacetonitriles, haloacetamides, halonitromethanes, haloketones, and unknown halogenated organic compounds, which can comprise more than 50% of the TOX [1,2,5,45].

This study frequently detected both urinary MCAA and TCAA (Table 2 and Table 3). Although urinary TCAA is being used as a biomarker of HAAs [41], the use of urinary MCAA as a possible biomarker has never been explored. The frequent detection rate of urinary MCAA (44%) and moderate correlation between urinary MCAA and TCAA (r = 0.45; *p* = 0.04) in this study suggest that MCAA along with TCAA may be used as potential biomarkers of HAA exposure. Because exposure routes and half-lives of individual HAAs may influence the exposure variability in humans, both cross-sectional and 24-h longitudinal analysis of measured HAAs are informative. The cross-sectional analysis of urinary MCAA and TCAA across participants indicates greater exposure variability in urinary MCAA (variance = 0.97) than urinary TCAA (variance = 0.70). The detected median TCAA level (2.5 μg/L) was comparable with urinary levels reported in the Canadian and US studies. In the Canadian study, the reported median TCAA level was 3.5 μg/L, while in the US study, the reported median TCAA level was 3.3 μg/L [41,55]. However, the greater exposure variability of may be associated with a relatively short half-life of MCAA (few hours) in the body compared to TCAA (1–6 days) [42,43]. In addition, other miscellaneous sources of MCAA may contribute to high exposure variability of urinary MCAA. MCAA is formed in the food production and processing industry through disinfection of poultry and meats, and sanitization of equipment and containers. Thus MCAA can be found in meat and other food products, including some vegetables, fruits, grains, and beer [56], and thus indicates that food in addition to drinking water may be an important exposure source of MCAA. However, the data are not adequate to quantify the contributions of each source for an overall assessment of exposure [57].

Animal studies on metabolic pathways suggest that the majority of MCAA is excreted as nonmetabolized MCAA. A minor fraction of MCAA reacts with glutathione and is excreted in urine as the conjugate. Mice excreted 80–90% of administered MCAA in their urine and 8% via exhaled air as CO_2_ within 24 h [58,59]. Similarly, TCAA is poorly metabolized in the liver of rats and mice, with no detectable metabolites (e.g., oxalate, glyoxalate, glycolate, or dichloroacetic acid) reported [17]. Moreover, Kim and Weisel (1999) showed that four human subjects dermally exposed to TCAA (and DCAA) showed no significant increase in urinary TCAA levels [42]. Unlike MCAA and TCAA, the pharmacokinetic studies in Fischer 344 rats indicate that the majority of DCAA metabolizes into other byproducts, and very little is excreted as unchanged DCAA in urine [60,61]. In a human study, intravenous or oral intake of DCAA in 12 healthy human volunteers (eight men and four women, aged 18–45 years) showed <1% urinary elimination of DCAA [62]. The liver plays a major role in the metabolism of DCAA, metabolizing DCAA into urinary glycolic acid, glyoxylic acid, and oxalic acid. Also, a fraction of DCAA is eliminated through exhalation as carbon dioxide, which further reduces the residual of DCAA in the body [60,61].

Among the brominated acetic acids, DBAA and unregulated BCAA were detected in all drinking water specimens, but no detectable levels were found in urine specimens. Similar to other dihaloacetic acids, both DBAA and BCAA appear to be metabolized into different byproducts. Limited number of in vitro pharmacokinetic studies suggest that DBAA and BCAA are metabolized to glyoxylic acid in a manner similar to DCAA [63,64]. Urine and feces are not the major contributors of overall elimination, suggesting that liver biotransformation was responsible for the rapid disappearance of DBAA and BCAA.

Our urinary PS data indicates that MCAA represents fairly recent exposure to drinking water HAAs, thus suggesting a weakness in most exposure and epidemiologic studies, which collect MV urine for measuring DBP levels [65,66]. The 24-h analysis of urinary MCAA and TCAA in this study demonstrates that MV urine does not reflect peak exposure to HAAs. MCAA and TCAA levels generally peak during daytime, primarily PS (generally between 0.75 and 3 hours after) or after other recent water use activity [65,66,67,68].

A review of toxicokinetic studies on MCAA indicates that humans are exposed to MCAA primarily through ingestion and dermal routes. MCAA is readily absorbed after ingestion [69], and through the skin [70,71]. Despite the low octanol–water partitioning coefficient (LogKow) of MCAA, i.e., 0.22, it is suspected to be absorbed by the dermal route in humans. Saghir and Rozman reported that most of the dermal MCAA dose rapidly penetrated into the skin (>95% within 0.25 h). MCAA concentrations in blood peaked at 0.36% of the dose by 0.75 h and remained constant for up to 4 h. Within 0.75 h, the liver had metabolized 9% of the dermally-absorbed MCAA, and participants had excreted ~64–72% of the dose [59]. Moreover, studies of human acute poisoning following dermal exposure to MCAA via accidental exposure (up to 90% MCAA) have been reported in the literature, which suggest that MCAA is rapidly absorbed and systemically distributed following direct skin contact covering at least 10% of the skin surface [70,71,72]. Although these studies represent acute or high dose exposure scenarios, which are not common in the general population through daily use of disinfected water, they do suggest that humans are susceptible to dermal exposure. Since MCAA is relative non-volatile, inhalation exposure to MCAA is limited through respiratory uptake of shower-generated aerosols. Weisel et al. (2003) found that average daily doses of aerosol HAAs from showering were 0.4–1.0 μg/day when water HAA concentrations were ~250 μg/L, suggesting that inhalation exposures to aerosolized MCAA during showering does not contribute significantly to the total exposure [65,66]. However, it is impossible to determine the contribution of inhalation route in this study because no air (aerosol) specimens were collected during shower activity.

Trend analysis showed higher exposure variability in urinary MCAA than urinary TCAA (Figure 2). Due to this variability in urinary MCAA and different possible exposure routes, the use of MCAA as a biomarker of HAAs is contingent upon the scope of the study design. MCAA can be a good biomarker of recent intake of disinfected water through ingestion and dermal uptake to capture short-term (acute) exposure. On the contrary, TCAA is much more stable over time and shows lower exposure variability when compare to urinary MCAA. Few biomonitoring studies have reported high exposure variability of urinary TCAA across individuals with different exposure levels [43,73,74]. Kim et al. (1999) reported low interindividual variability in urinary excretion rates of TCAA with ingestion of chlorinated water [42]. TCAA is a major nonvolatile DBP of water disinfection, and hence inhalation exposure is minimal compared to ingestion. Also, dermal exposure of TCAA shows no significant increase in urinary TCAA levels [42,65]. Thus, TCAA is a suitable biomarker of long-term (chronic) exposure through the ingestion route. However, because the water consumption pattern of an individual varies considerably within and across days, the collection of multiple specimens will still be preferred for reliable exposure measures [41].

Unlike few other studies, our study reported a moderate but statistically insignificant correlation between urinary and drinking water TCAA (r = 0.52; *p* = 0.07). Zhang et al. (2009) reported comparable correlations between TCAA from ingestion of drinking water and urinary excretion of TCAA (r = 0.66; *p* < 0.001) [41]. Smith et al. (2013) showed correlations between urine and drinking water HAAs for employed women with different water sources (r = 0.31, *p* = 0.20) and unemployed women with one water source (r = 0.44, *p* = 0.007) [75]. Individual tap water intake is effective for estimating TCAA levels because water ingestion at home is a valid proxy for TCAA exposure for unemployed women with no apparent change in source water, but is less satisfactory for employed women with multiple potential water sources. Similar findings are reported in other studies where TCAA excretion in urine did not correlate significantly with measured water concentrations of TCAA [42,65,66]. In our study, the insignificant correlation between urine and drinking water TCAA is suspected due to differences in water consumption pattern, behavioral differences, and potentially multiple water sources. Although, all the participants consumed unfiltered residential tap water during sampling period, they reported intermittent water consumption from other sources (e.g., gym, public park, trail, office, bottle water, soda, juices, milk, coffee, and tea). Due to small number of participants and voluntary nature of reporting water consumption pattern and behavioral activities, we did not have adequate data for further analysis. We anticipate that TCAA in drinking water should be a good predictor of urinary TCAA when individual-level water consumption data (frequency and quantity) and source information (e.g., bottled water, office, home, and gym) are available. Most exposure and epidemiologic studies failed to collect this granular level of information from each participant [9,27,35,36,37,38,76,77]. We did as well, and hence, it is not surprising that the TCAA drinking water levels did not correlate with urinary TCAA.

### Key Limitations and Future Considerations

This study provides preliminary but direct measures of HAAs. Both longitudinal and cross-sectional study designs and use of urinary measures provide novel short-term exposure data on individual HAAs and their association with exposure routes. This information is critical in the designing of future exposure investigations for reliable exposure estimates for health risk assessment applications, particularly reproductive health risk (e.g., adverse birth outcomes). However, these findings are suggestive due to the exploratory nature of this study. In addition to the small scale of this study, the user-linked behavioral data on comprehensive exposure activities were limited. For example, no data were collected on miscellaneous water sources, shower duration, water temperature during shower, air (aerosol) concertation of HAAs, volume of water intake, and physical activities. These are important considerations for evaluating the overall and route-specific contribution of urinary HAAs. Similarly, our trend analysis suggests that the dermal and inhalation routes are prominent exposure routes of urinary MCAA, but we did not have any direct measures of dermal uptake and inhalation intake to confirm these findings. Also, our trend analysis was based on three participants with single measurement per event (MV, PS, and NT), which were inadequate to test for significant exposure variability across repeat measures. Therefore, a large follow-up study is warranted for reproducibility of these results in other water systems, population type (e.g., pregnant women), and exposure scenarios. These limitations should be considered at the planning stage of future investigations for a comprehensive of water related exposure activities and environmental variables (e.g., season, temperature, and ventilation) for an improved exposure characterization. Despite above discussed limitations, the findings from this study are novel and informative for the exposure scientists to further built upon to characterize urinary HAAs for health risk assessment.

## 5. Conclusions

This study reports for the first time the detection of MCAA and TCAA in both longitudinally and cross-sectionally collected human urine specimens. Both MCAA and TCAA were frequently detected and showed moderate correlations, suggesting that MCAA and TCAA can be used as biomarkers of HAA exposure. Urinary MCAA appears to be a reliable biomarker of recent intake of chlorinated or chloraminated drinking water through dermal and inhalation routes, while urinary TCAA is a reliable biomarker of chronic exposure through the ingestion route. On the contrary, drinking water HAAs do not appear to give reliable measures of human exposure. Hence their use as exposure surrogates should be avoided unless individual-level data on water consumption, frequency of intake, and water-source-specific information are available. The findings from this study will inform future exposure and epidemiologic investigations of halogenated DBPs. However, further research is needed to understand the exposure behavior and toxicokinetic mechanisms of MCAA and TCAA, especially their half-lives and inhalation route specific contribution to total exposure.

## Figures and Tables

**Table 1:** Participants recruitment and specimen collection plan at Site A and B.

Site	Number of Participants	Specimen Type	Specimens	
			Longitudinal	Cross-Sectional
Site-A	7	UR	11	18
		DW		7
Site-B	10	UR		10
		DW		10

Legend: UR represents urine specimen; DW represents drinking water specimen. Longitudinal (24-h) urinary specimens were collected from Site A only. One water specimen was collected from each participant.

**Table 2:** Measured concentrations (in µg/L) of HAAs in urine specimens.

HAAs	N (%)	Q1	Q2	Q3	Q4	IQR	Mean	SD	Min	Max	CV	<LOD (%)
MCAA	20 (100)	1.0	1.0	4.6	10.7	3.6	3.2	3.1	1.0	12.6	0.97	11 (56)
Site A	10 (50)	1.0	1.0	4.3	6.3	3.3	2.6	2.1	1.0	6.3	0.8	6 (59)
Site B	10 (50)	1.0	1.7	4.7	10.7	3.7	3.7	3.8	1.0	10.7	1.0	5 (50)
TCAA	20 (100)	1.6	2.5	3.9	7.6	2.3	2.9	2.0	0.5	7.6	0.7	3 (11)
Site A	10 (50)	2.2	2.5	3.5	7.0	1.3	3.1	1.6	1.6	7.0	0.5	0 (0)
Site B	10 (50)	0.6	2.3	3.9	7.6	3.2	2.7	2.3	0.5	7.6	0.9	3 (30)

Legend: N represents the number of data points that were used to generate summary statistics. Although, a total of 18 urinary specimens were collected from Site-A (11 longitudinal specimens and 7 cross-sectional specimens), we used only three mean data points from the longitudinal study (mean values of participants A1, A2, and A3), and all seven data points from the cross-sectional study for summary statistics in Table 2. All individual level data on the cross-sectional and longitudinal studies are provided in the supporting material section-Table S1. Since longitudinal and cross-sectional studies were conducted 2-weeks apart, the three mean values from the longitudinal study and seven single measures from the cross-sectional study were treated as independent and mutually exclusive data points for summary statistics. We did not use all individual measures from the longitudinal study to generate summary statistics because it potentially violates the assumption of mutual independence due to same day specimen collection. IQR shows interquartile range of individual quartiles at 25^th^ (Q1), 50^th^ (Q2), 75^th^ (Q3), and 95^th^ (Q4); SD represents standard deviation in the measured levels; CV indicates coefficient of variance; and LOD represents the limit of detection. LODs for DCAA, TCAA, DBAA, and BCAA were 1 µg/L, and MCAA was 2 µg/L. DCAA, DBAA, MBAA, and BCAA were below LOD in all urinary specimens. MCAA and TCAA values that were below LOD were replaced with LOD/2. A similar summary data analysis of measured urine haloacetic acids (HAAs) was performed excluding urine data below LOD to determine any significant change in median values. Table S2 is provided in the supporting material section to show the summary of measured concentrations of HAAs in urine specimens without nondetects. The difference between MCAA and TCAA levels measured at Site A and B remained statistically insignificant without nondetects (MCAA *p* = 0.58; and TCAA *p* = 0.49).

**Table 3:** Measured concentrations (in µg/L) of HAAs in water specimens.

HAAs	N (%)	Q1	Q2	Q3	Q4	IQR	Mean	SD	Min	Max	CV
DCAA	17 (100)	14.8	17.4	18.8	20.9	4.0	15.8	4.9	2.3	20.9	0.3
Site A	7 (41)	9.4	14.8	16.1	17.4	6.7	12.2	5.8	2.3	17.4	0.5
Site B	10 (59)	17.5	18.7	19.4	20.9	1.9	18.4	1.8	14.8	20.9	0.1
TCAA	17 (100)	6.5	7.4	7.9	11.0	1.4	6.6	3.4	0.5	11.0	0.5
Site A	7 (41)	0.9	2.9	10.7	11.0	9.8	5.4	5.1	0.5	11.0	1.0
Site B	10 (59)	7.1	7.5	7.6	9.6	0.4	7.5	0.9	6.5	9.6	0.1
DBAA	17 (100)	0.5	0.5	0.5	1.8	0.0	0.6	0.4	0.5	1.8	0.6
Site A	7 (41)	0.5	0.5	1.1	1.8	0.6	0.8	0.6	0.5	1.8	0.7
Site B	10 (59)	0.5	0.5	0.5	0.5	0.0	0.5	0.0	0.5	0.5	0.0
BCAA	17 (100)	3.7	4.4	4.7	4.9	1.0	4.2	0.7	2.2	4.9	0.2
Site A	7 (41)	3.5	3.6	4.3	4.7	0.8	3.7	0.9	2.2	4.7	0.2
Site B	10 (59)	4.4	4.5	4.7	4.9	0.3	4.5	0.3	3.7	4.9	0.1

Legend: N represents the total number of drinking water specimens collected from Sites A and B. MCAA and MBAA were below LOD in all drinking water specimens. Limits of detection for MCAA and MBAA were 1 µg/L and 2 µg/L, respectively. The difference in measured levels at Sites A and B was significant for DCAA (*p* = 0.01) and insignificant for TCAA (*p* = 0.66), DBAA (*p* = 0.10), and BCAA (0.13).

## Supplementary Materials

The following are available online at http://www.mdpi.com/1660-4601/16/3/471/s1, Table S1: Summary data on individual measurement of HAAs in urine and drinking water specimens, Table S2: Summary statistics of urinary MCAA and TCAA (μg/L) without data below limit of detection (LOD), Table S3: Intraday change in urinary MCAA and TCAA levels (μg/L).

## Abbreviations

BCAA	bromochloroacetic acid
CV	coefficient of variance
DBAA	dibromoacetic acid
DBPs	disinfection byproducts
DCAA	dichloroacetic acid
EPA	Environmental Protection Agency
GSH	glutathione
HAAs	haloacetic acids
IQR	inter-quartile range
LOD	limit of detection
MBAA	monobromoacetic acid
MCAA	monochloroacetic acid
NOM	natural organic matter
Q1	first quartile between 0^th^-25^th^ percentile
Q2	second quartile between 25^th^-50^th^ percentile
Q3	third quartile between 50^th^-75^th^ percentile
Q4	second quartile between 75^th^-99^th^ percentile
SD	standard deviation
TCAA	trichloroacetic acid
THMs	trihalomethanes
TOBr	total organic bromine
TOCl	total organic chlorine
TOI	total organic iodine
TOX	total organic halogen
