# Supplementary material for: Exposure Characterization of Haloacetic Acids in Humans for Exposure and Risk Assessment Applications: An Exploratory Study
Source: Int J Environ Res Public Health. 2019 Feb 6;16(3):471. doi: 10.3390/ijerph16030471 (PMC6388255; doi:10.3390/ijerph16030471)
Supplement: Supplementary file 1 [file ijerph-16-00471-s001.pdf]

Table S1. Summary data on individual measurement of HAAs in urine and drinking water specimens.

			Urine (µg/L)						Drinking water (µg/L)					
SITE	Participant ID	Sample ID	MCAA	DCAA	TCAA	MBAA	DBAA	BCAA	MCAA	DCAA	TCAA	MBAA	DBAA	BCAA
Site-A	A1	A1a	<2	<1	1.284	<1	<1	<1						
Site-A	A1	A1b	8.56	1.018	2.14	<1	<1	<1						
Site-A	A1	A1c	10.28	<1	2.8	<1	<1	<1						
Site-A	A1	A1d	<2	<1	1.34	<1	<1	<1						
**Site-A**	**A1**	**[#]Mean**	**5.21**	**0.630**	**1.891**	**0.5**	**.05**	**0.5**						
Site-A	A2	A2a	9.5	<1	2.88	<1	<1	<1						
Site-A	A2	A2b	4.22	<1	2.38	<1	<1	<1						
Site-A	A2	A2c	<2	<1	1.016	<1	<1	<1						
Site-A	A2	A2d	<2	<1	4.28	<1	<1	<1						
**Site-A**	**A2**	**[#]Mean**	**3.93**	**0.5**	**2.639**	**0.5**	**0.5**	**0.5**						
Site-A	A3	A3a	4.02	<1	4.76	<1	<1	<1						
Site-A	A3	A3b	12.6	<1	5.4	<1	<1	<1						
Site-A	A3	A3c	2.2	<1	3.5	<1	<1	<1						
**Site-A**	**A3**	**[#]Mean**	**6.273**	**0.5**	**4.553**	**0.5**	**0.5**	**0.5**						
Site-A	A1	A1-1	<2	<1	2.26	<1	<1	<1	<2	14.8	<1	<1	<1	3.62
Site-A	A2	A2-1	4.44	<1	2.7	<1	<1	<1	<2	14.6	<1	<1	<1	3.60
Site-A	A3	A3-1	<2	<1	2.28	<1	<1	<1	<2	17.4	11	<1	<1	4.7
Site-A	A4	A4-1	<2	<1	7	<1	<1	<1	<2	15.1	<1	<1	<1	3.5
Site-A	A5	A5-1	<2	<1	2.16	<1	<1	<1	<2	6.11	2.91	<1	1.6	3.4
Site-A	A6	A6-1	<2	<1	3.82	<1	<1	<1	<2	2.25	1.24	<1	1.79	2.15
Site-A	A7	A7-1	<2	<1	1.628	<1	<1	<1	<2	12.6	10.3	<1	<1	3.91
Site-B	B1	B1-1	2.4	<1	3.52	<1	<1	<1	<2	19.4	7.42	<1	<1	4.62
Site-B	B2	B2-1	<2	<1	4	<1	<1	<1	<2	20.2	7.91	<1	<1	4.67
Site-B	B3	B3-1	<2	<1	1.032	<1	<1	<1	<2	18.8	6.67	<1	<1	4.42
Site-B	B4	B4-1	<2	<1	1.508	<1	<1	<1	<2	19.3	6.47	<1	<1	4.36
Site-B	B5	B5-1	<2	<1	<1	<1	<1	<1	<2	17.3	7.08	<1	<1	4.48
Site-B	B6	B6-1	<2	<1	3	<1	<1	<1	<2	20.9	7.51	<1	<1	4.36
Site-B	B7	B7-1	10.38	<1	7.64	<1	<1	<1	<2	14.8	9.61	<1	<1	3.66
Site-B	B8	B8-1	4.96	<1	<1	<1	<1	<1	<2	16.8	7.31	<1	<1	4.51
Site-B	B9	B9-1	3.72	<1	<1	<1	<1	<1	<2	18.5	7.56	<1	<1	4.94
Site-B	B10	B10-1	10.7	<1	4.54	<1	<1	<1	<2	18	7.5	<1	<1	4.69

Legend: The limit of detection for DCAA, TCAA, MBAA, DBAA, and BCAA were 1 µg/L, and MCAA was 2 µg/L. ParticipantA1, A2, A3 provided both longitudinal and cross-sectional specimens. First 11 specimens in the table show longitudinal specimens from the participants A1, A2, and A3.[#]Indicate the mean values of longitudinal specimens collected from participant A1 (sample ID-A1a, A1b, A1c, A1d), A2 (sample ID-A2a, A2b, A2c, A2d), and A3 (sample ID-A3a, A3b, A3c). These mean values and seven cross-sectional measures from site-A were used to generate summary statistics in Table 2a. Since longitudinal and cross-sectional studies were conducted two-weeks apart, the mean values of participant A1, A2, and A3 in the longitudinal study and single measures from the cross-sectional study were treated as independent and mutually exclusive for summary statistics. We did not use all individual measures from the longitudinal study to generate summary statistics because it potentially violates the assumption of mutual independence due to same day specimen collection.

Table S2. Summary statistics of urinary MCAA and TCAA (μg/L) without data below limit of detection (LOD).

HAAs	N	Q1	Q2	Q3	Q4	IQR	Mean	SD	Min	Max	CV
MCAA	9	4.44	6.27	9.42	10.70	4.98	6.57	3.01	2.40	10.70	0.46
Site-A	4	5.82	6.57	7.50	9.42	1.68	6.75	3.86	4.44	9.42	0.57
Site-B	5	3.72	4.96	10.38	10.70	6.66	6.43	3.86	2.40	10.70	0.60
TCAA	17	2.16	2.70	4.00	7.64	1.84	3.30	1.84	1.03	7.64	0.56
Site-A	10	2.19	2.46	3.54	7.00	1.35	3.09	1.64	1.63	7.00	0.53
Site-B	7	2.25	3.52	4.27	7.64	2.02	3.61	2.19	1.03	7.64	0.61

Legend: 'N' represents the total number of urinary specimens with detected levels of urinary MCAA and TCAA; IQR shows interquartile range of individual quartiles at 25th (Q1), 50th (Q2), 75th (Q3), and 95th (Q4); SD represents standard deviation in the measured levels; CV indicates coefficient of variance. The difference between MCAA and TCAA levels measured at Site A and B remained statistically insignificant without non-detects (MCAA p=0.90; and TCAA p=0.67).

Table S3. Intraday change in urinary MCAA and TCAA levels (μg/L).

	MCAA			TCAA		
Participant ID	MV	PS	NT	MV	PS	NT
A1	8.6	10.3	1.0 and 1.0	2.1	2.8	1.3 and 1.3
A2	4.2	9.5	1.0 and 1.0	2.4	2.9	1.0 and 4.3
A3	4.0	12.6	2.2	4.8	5.4	3.5

Legend: MV presents the morning void specimen; PS represents the post-shower specimen; NT represents the night time specimen. Participants A1, A2, and A3 provided intraday urinary specimen from Site-A only. All measurements were based on single specimen per participant with an exception of NT specimens from participant A1 and A2 who provided two NT specimens.
